# Horizontal equity in access to public GP services by socioeconomic group: potential bias due to a compartmentalised approach

**DOI:** 10.1186/s12939-019-1091-2

**Published:** 2019-12-02

**Authors:** M. A. Negrín, J. Pinilla, I. Abásolo

**Affiliations:** 1 Departamento de Métodos Cuantitativos en Economía y Gestión, Universidad de Las Palmas de Gran Canaria, Las Palmas, Spain; 2 Departamento de Economía Aplicada y Métodos Cuantitativos. Instituto Universitario de Desarrollo Regional, Universidad de La Laguna, Facultad de Economía, Empresa y Turismo, Campus de Guajara, 38071 La Laguna, Santa Crus de Tenerife Spain

**Keywords:** Public GP visits, Horizontal equity in access, Bias, Health care services, Specialist visits, National health surveys

## Abstract

**Background:**

Horizontal equity in access to public general practitioner (GP) services by socioeconomic group has been addressed econometrically by testing the statement “equal probability of using public GP services for equal health care needs, regardless of socioeconomic status”. Based on survey data, the conventional approach has been to estimate binomial econometric models in which when the respondent reports having visited a public GP, it counts as 1, otherwise it counts as 0. This is what we call a *compartmentalised approach*. Those respondents who did not visit a public GP but visited instead another doctor (specialist or private GP) would count as 0 (despite having used instead other modes of health care), thus conclusions of the compartmentalised approach might be biased. In such cases, a multinomial econometric model -that we called *comprehensive approach*- would be more appropriate to analyse horizontal equity in access to public GP services. The objective of this paper is to test for this potential bias by comparing a *compartmentalised* and a *comprehensive* approach, when analysing horizontal equity in access to public GP.

**Methods:**

Using data from the 2016/17 Spanish National Health Survey, we estimate the probability of visiting a public GP as determined by socioeconomic status, health care need and demographic characteristics. We use binomial and multinomial logit and probit models in order to highlight the potential differences in the conclusions regarding socioeconomic inequities in access to public GP services. Socioeconomic status is proxied by education level, social class and employment situation.

**Results:**

Our results show that conclusions are sensitive to the approach selected. Particularly, the horizontal inequity favouring individuals with lower education that resulted from the compartmentalised approach disappears under a comprehensive approach and only a social class effect remains.

**Conclusion:**

An analysis of horizontal equity in access to a particular health care service (like public GP services) undertaken following a compartmentalised approach should be compared with a comprehensive approach in order to test that there is no bias as a consequence of considering as zeros the utilisation of other types of health care.

## Introduction

There is ample literature in health economics that analyses horizontal inequities in access to public sector general practitioner (GP) services by socioeconomic groups following an econometric approach. In this paper we refer to those studies that -using survey data- test the principle of “equal probability of visiting a GP for equal health care needs” by means of econometric models. Generally, these studies estimate the probability of visiting a GP in a particular period, as a function of health care need and other socioeconomic and demographic characteristics of the individual. Socioeconomic status is generally approached by education, social class or/and income.

Most of these studies show evidence of a gradient in the probability of using GP services favouring those in lower socioeconomic status. For example, Abásolo et al. [1] analyse horizontal equity in access and utilisation of public GP services in Spain applying probit models to data from the Spanish national health survey of 1993. They find evidence of horizontal inequity that favours men and women in lower socio-economic groups, women with fewer educational qualifications and men who are not working. Urbanos [2] uses the Spanish national health surveys of 1987, 1993 and 1995 and probit models to explain the inequality in the use of public health care services among individuals with similar medical needs. For the case of public GP services, she concludes that in general, more highly educated individuals are more likely to under-consume compared to the population whose education is minimal. In addition, her results indicate that the propensity towards excessive consumption increases as social class decreases, except for the year 1995. Morris et al. [3] investigate inequity in the use of general practitioner consultations and other health care services of the National Health Service in England. Their analysis is based on pooled data from three rounds (1998, 1999, 2000) of the English health survey. They use linear probability models to estimate the probability of visiting a GP finding evidence that those with lower education attainment are more likely to visit their GP. They also find that income (and being unemployed) is negatively correlated with use. Regidor et al. [4] study horizontal equity in the utilisation of public GP services in Spain using data from the 2003 Spanish national health survey. They estimate the probability of visiting a GP through a binomial regression considering education level and social class variables among covariates. They show the existence of inequity in public GP visits favouring individuals with lower education levels and also those belonging to lower social classes. Abásolo et al. [5] using the national health survey of 2006 and through multilevel binary logistic functions, analyse horizontal equity in the probability of utilisation of public health care services in Spain by socioeconomic groups. They find that education level is negatively related to the probability of visiting a public GP. A negative correlation is also found for income and for being employed. González and Clavero [6] analyse, through dynamic models, the explanatory factors of income related inequality in health care utilisation in Spain (they consider only those patients covered by the public health care system, excluding those individuals with private insurance). They use the European Community Household Panel. They find that individuals with higher income have a lower probability to visit a GP; in addition, education is the main determinant of the inequality of utilisation as those with primary studies or less have a higher probability to visit a GP. Similar conclusions are reached by Gonzalez and Clavero [7] in another study with the same data base in which they study the inequalities in health care utilisation caused by the double health insurance coverage in Spain.

Other studies show no evidence of horizontal inequity by socioeconomic status or show horizontal inequity favouring the most advantaged. For example, Van Der Heyden et al. [8], with data from the 1997 Belgian National Health Interview Survey, used logistic regressions to adjust by demographic and health status variables (in addition to socioeconomic variables), finding no evidence of inequity in the probability to use GP services by education or income variables. San Sebastian et al. [9] use the 2006, 2010 and 2014 cross sectional household surveys (“health on equal terms surveys”) for the four northern-most counties in Sweden. Using probit regressions and analysing and computing the horizontal inequity indexes find a pro-rich inequality in access to GPs, particularly for 2010 and 2014, being the most important contributor income and education, attributable -they argue- to underutilisation among those with lowest education. Wagenius et al. [10] use the same data base than [9] but just for 2014, selecting young respondents -between 16 and 24 years-. They undertake binomial regressions to explain the probability of visiting the GP, youth clinics and nurses. They found pro-poor inequity in access to GP services both for men and women. However, they also found a higher probability of using GP services for those with higher education levels for men, but not for women for whom there is no evidence of horizontal inequity.

A common characteristic in all of the above studies is that they use binomial models (eg. logit, probit, linear probability models). That is, the dependent variable is a binary variable, so that if a respondent reports that she visited a public GP, then it counts as 1, otherwise it counts as zero. In addition, when these studies analyse also the equity in the utilisation of other health care services (specialist, GP, emergency services, etc.), these are considered separately, in different binomial models, independent of each other. That is, all the above studies take a *compartmentalized approach* to the problem of horizontal equity in access to health care services. However, in the analysis of equity in access to a particular service (eg. public GP service) it may be the case that the respondent reports to have used the health care system, but instead of visiting a public GP, she was in another point of the public health care system (i.e. visiting a specialist doctor) or was voluntarily visiting a private doctor. Binomial econometric models for equity in access to public GP services would also consider such visits as zeros (i.e. non-utilisation), thus potentially obtaining biased conclusions regarding horizontal equity in access. With the aim to test for this potential bias, the objective of this research is to study whether the *compartmentalized approach* to the problem of horizontal equity in access to public GP services reaches the same conclusions as a *comprehensive approach* in which, in addition to adjusting for variables of health care need and other demographic and socioeconomic characteristics, we also consider the use of other public and private health care services, thus avoiding an inappropriate (we believe) treatment of zeros. We undertake this analysis using the same data base (the Spanish National Health Survey 2016–17) in order to maximise comparability of the two approaches. To the best of our knowledge, this exercise has not been carried out in the related literature so far. In what follows, Section 2 presents the data, variables and methods used. The results are presented in Section 3 and discussed in Section 4. Conclusions are presented in Section 5.

## Data, variables and methods

### Data and variables

The data used in this research come from the 2016–17 Spanish National Health Survey. This is a face-to-face, cross-sectional population-based survey that employs a three-stage, stratified-random design to identify samples of adults (aged 15 or over) within the target number of survey interviews (23,089). The first-stage units are the census sections, which are stratified according to the size of the municipality to which the section belongs. The second-stage units are the main households. The third stage units are chosen from a list of persons within the household who can be interviewed and asked to fill in the questionnaire at the time the survey is carried out (for more details, see [11]). Data on health care utilisation, self-reported morbidity and other demographic and socioeconomic characteristics are also collected.

On the one hand, for the compartmentalised approach, the dependent variable of interest is a dummy variable representing whether or not the individual has visited a public sector GP in the past four weeks. On the other hand, for those who report not having visited a public GP, we have also considered whether they have visited instead a public specialist doctor or a private doctor (specialist or general practitioner); thus for the comprehensive approach the dependent variable refers to whether or not the individual has visited one of the four different health services. The type of visit -public or private sector visit, and whether it was a GP visit or a specialist visit- is only known for the last visit of the respondent and we focus our study on this last visit. Therefore, the different outcomes of the dependent variables have been built upon the combination of four survey questions. Firstly, respondents are asked whether they have visited a GP in the past four weeks. Secondly, respondents are asked whether they have visited a specialist doctor in the past four weeks. Thirdly those who have visited both sorts of doctors in the same period are asked the nature (GP or specialist) of the very last visit. Finally, individuals are asked whether the doctor they had consulted the last time was in the public health system, was from a private insurance company or was in a private consultation. We considered the former case as a public visit, whilst the second and third cases were considered as private visits. Unfortunately, information about other health care services like emergency services is referred to a different period (12 months) so it has not been possible to take them into account in the analysis.

Regarding the explanatory variables, socioeconomic status (main focus of this paper) is proxied through three variables: education, social class and employment situation. Education is measured by a categorical variable indicating the highest level of education achieved by the respondent: no studies, primary studies, secondary studies and university studies. Social class of the reference person in the household is also measured by a categorical variable with four categories based on the National Classification of Occupations: high social class (Directors and managers with university degrees), medium-high social class (intermediate professions and self-employed), medium-low social class (skilled and partly-skilled occupations), and low social class (unskilled workers). Employment situation of the respondent is measured by a categorical variable with three possible activity statuses: employed (the individual is currently employed), unemployed (the individual is currently unemployed) or inactive (the individual is retired, disabled or housemaker). We have not included an income variable due to the number of missing cases (24% in the Spanish National Health Survey of 2017), so for the purpose of this paper, we considered sufficient the three proxies for socioeconomic status included in the analysis.

In order to adjust by health care need, we have considered self-reported measures of individuals’ health state. These include a categorical indicator that records whether individuals considered their general health during the twelve months prior to the survey to be ‘very good’, ‘good’, ‘fair’, ‘poor’ or ‘very poor’. We have also considered a dummy variable indicating whether the respondent reports the presence of any chronic conditions. Regarding demographic characteristics, we have included age and sex of the respondents.

### Methods

- Binary models for a compartmentalized approach.

In a compartmentalized approach, the dependent variable (Y) is a binary variable with only two possible values: 1 if the individual has used the public GP service, and 0 otherwise. The binary response models most commonly used are the logit and probit models [12].

Both models assume that the conditional probability of Y = 1 given a vector of covariates x is:

$$ P\left(Y=1|x\right)=F\left(x^{\prime}\beta \right) $$

where β is the vector of parameters of the model. A binary response model is referred to as a logit model if *F* is the cumulative logistic distribution function, and it is called a probit model if *F* is the cumulative normal distribution function. Both logit and probit models can also be described as latent variable models, where there is an auxiliary random variable *Y*^∗^ = *x* ′ *β*, where the dependent variable Y can be viewed as an indicator for whether this latent variable is positive (Y = 1 if *Y*^∗^ > 0 and 0 otherwise):

$$ \mathrm{logit}\ \mathrm{model}:\frac{P\left(Y=1|x\right)}{1-P\left(Y=1|x\right)}={e}^{x\prime \beta } $$

probit model : *P*(*Y* = 1| *x*) = *P*(*Y*^∗^ > 0) = Φ(*x* ′ *β*)

The logistic and normal distributions are both symmetrical around zero, but the logistic distribution has fatter tails. Both methods yield similar inferences, although logit models have become more popular in social sciences because coefficients can be interpreted in terms of odds ratios.

- Multinomial models for a comprehensive approach.

In a comprehensive approach, the dependent variable is a nominal variable with five categories, indicating the health service used on the last visit (public or private GP or specialist), in addition to the possibility of not having used any service in the last four weeks. In this case, it is necessary to consider multinomial (logistic or probit) regressions [13, 14].

A multinomial logistic model for k possible outcomes can be described as a combination of (k-1) independent binary logit models, in which one outcome is chosen as a reference.

$$ \frac{P\left(Y=1|x\right)}{P\left(Y=k|x\right)}={e}^{x\prime {\beta}_1},\frac{P\left(Y=2|x\right)}{P\left(Y=k|x\right)}={e}^{x\prime {\beta}_2},\dots, \frac{P\left(Y=k-1|x\right)}{P\left(Y=k|x\right)}={e}^{x\prime {\beta}_{k-1}} $$

Since the k probabilities must sum to one, it is straightforward to obtain their expressions:

$$ P\left(Y=1|x\right)=\frac{e^{x\prime {\beta}_1}}{1+\sum \limits_{j=1}^{k-1}{e}^{x\prime {\beta}_j}},P\left(Y=2|x\right)=\frac{e^{x\prime {\beta}_2}}{1+\sum \limits_{j=1}^{k-1}{e}^{x\prime {\beta}_j}},\dots, $$

$$ P\left(Y=k-1|x\right)=\frac{e^{x\prime {\beta}_{k-1}}}{1+\sum \limits_{j=1}^{k-1}{e}^{x\prime {\beta}_j}},P\left(Y=1|x\right)=\frac{1}{1+\sum \limits_{j=1}^{k-1}{e}^{x\prime {\beta}_j}} $$

The multinomial logit relies on the assumption of independence of irrelevant alternatives (IIA), which states that the odds of preferring one alternative over another do not depend on the presence of other alternatives. Multinomial probit can be used as an alternative when IIA assumption is violated.

The multinomial probit model can be described in terms of a latent variable model. The latent variables for each alternative are ${Y}_1^{\ast }=x^{\prime }{\beta}_1+{\varepsilon}_1$, ${Y}_2^{\ast }=x^{\prime }{\beta}_2+{\varepsilon}_2$, …, ${Y}_k^{\ast }=x^{\prime }{\beta}_k+{\varepsilon}_k$, where *ε* = (*ε*_1_, *ε*_2_, …, *ε*_*k*_) follows a multivariate normal distribution with mean vector zero and a specified covariance matrix. Then

$$ Y=\left\{\ \begin{array}{c}1\kern0.75em \mathrm{if}\kern0.75em {Y}_1^{\ast }>{Y}_2^{\ast },\dots, {Y}_k^{\ast }\ \\ {}\begin{array}{c}2\kern0.75em \mathrm{if}\kern0.75em {Y}_2^{\ast }>{Y}_1^{\ast },\dots, {Y}_k^{\ast}\\ {}\dots \end{array}\\ {}k\kern0.75em \mathrm{if}\kern0.75em {Y}_k^{\ast }>{Y}_1^{\ast },\dots, {Y}_{k-1}^{\ast}\end{array}\right. $$

This model does not respect IIA, since error terms can be arbitrary correlated.

- An alternative Binomial model for a comprehensive approach.

A different way to address the existence of multiple alternatives of health service utilisation but without including them in the regression model would be to drop from the sample those individuals who in the last visit reported to use other health services different from public GP services (private GP services or private or public specialist services). Then a binomial probit or logit model would be estimated for the remaining sample of individuals. However, if individuals finally selected are not at random, this would lead to biased estimates. The probit model with sample selection [15] allows to test for selection bias. It considers two probit models: one for the probability of visiting a public GP (probit equation) and another one for the probability of being selected for the probit equation which, in our case is 1 minus the probability of visiting a doctor other than the public GP (selection equation). It is assumed that the error terms for both equations follow a bivariate normal distribution with a *rho* correlation. When *rho* is nonzero, the standard probit model applied to the probit equation yields biased results.

For the model to be well identified, the selection equation should have at least one variable that is not in the probit equation. For this reason, the selection equation includes a dummy variable for having double coverage, that is, individuals who have private health insurance in addition to public health care insurance, on the basis that having private insurance may explain participation (i.e. through the demand of private GP or specialist visits) but probably does not affect the probability to visit a public GP.

## Results

Table 1 shows the descriptive statistics for the dependent and independent variables. Regarding the dependent variable, 24.8% of individuals reported that the last visit they had in the past four weeks was made to a public GP. According to the compartmentalised approach, the remaining 75.2% would be considered as zeros in the binomial models. There were 12.1% of individuals who reported that the last visit they had in the past four weeks was to another professional, different from a public GP. Specifically, 7.7% visited a public specialist, 2.9% visited a private specialist and 1.5% visited a private GP (all of them referred to the last visit in the same 4-week period). According to the comprehensive approach, these 12.1% of individuals would not count as zeros (zeros would be the case in the remaining 63.1%, who did not visit a doctor at all).

**Table 1:** Descriptive stats

Type Var.	Variable	Mean	N
Dependent variable (last health service visit during the past four weeks)	Public primary care	0.2478	23,062
	Private primary care	0.0148	
	Public specialist care	0.0771	
	Private specialist care	0.0289	
	No visits	0.6314	
Demographic variables	Female	0.5411	23,089
	Age 15–34	0.1687	23,089
	Age 35–44	0.1797	
	Age 45–54	0.1782	
	Age 55–64	0.1693	
	Age more than 64	0.3042	
Health state variables	Health very good	0.1815	23,089
	Health good	0.4827	
	Health fair	0.2396	
	Health bad	0.0747	
	Health very bad	0.0216	
	Chronic	0.6926	23,081
Socioeconomic Variables	Primary studies or less	0.3121	23,089
	Obligatory Secondary studies	0.3062	
	Occupational training or pre-university studies	0.2002	
	University studies	0.1815	
	High social class (SC)	0.1803	22,483
	Medium-high SC	0.3359	
	Medium-low SC	0.3397	
	Low social class	0.1442	
	Inactive	0.4627	23,089
	Employed	0.4296	
	Unemployed	0.1077	

To begin with the compartmentalised approach, estimates for binomial logit and probit models are presented in Table 2. Sign and statistical significance of coefficients for both models are very similar. With respect to health care need variables, as expected, individuals who have a worse health state have a higher propensity of reporting visits to a public GP within the past four weeks, with a clear gradient as the state of health worsens. The probability of visiting a public GP is also greater for those individuals who suffer a chronic disease.

**Table 2:** Estimates of binomial logit and probit models

Variables	Binomial logit		Binomial probit	
	Coefficient (se)	*p*-value	Coefficient (se)	*p*-value
Female	0.1284 (0.0331)	0.000	0.0802 (0.0193)	0.000
Age 15–34	Ref.	Ref.	Ref.	Ref.
Age 35–44	−0.0742 (0.0647)	0.251	−0.0429 (0.0361)	0.234
Age 45–54	− 0.0701 (0.0635)	0.269	− 0.0413 (0.0357)	0.248
Age 55–64	0.0800 (0.0621)	0.197	0.0461 (0.0355)	0.194
Age more than 64	0.2863 (0.0661)	0.000	0.1756 (0.0382)	0.000
Health very good	Ref.	Ref.	Ref.	Ref.
Health good	0.2958 (0.0574)	0.000	0.1598 (0.031)	0.000
Health fair	0.8103 (0.0635)	0.000	0.4718 (0.0354)	0.000
Health bad	1.0785 (0.0768)	0.000	0.6399 (0.0446)	0.000
Health very bad	1.1264 (0.1092)	0.000	0.6694 (0.0656)	0.000
Chronic	0.6672 (0.0485)	0.000	0.3667 (0.0265)	0.000
Inactive	Ref.	Ref.	Ref.	Ref.
Employed	−0.1512 (0.0516)	0.003	−0.0871 (0.0299)	0.004
Unemployed	−0.0262 (0.0653)	0.688	−0.0163 (0.038)	0.669
Primary studies or less	Ref.	Ref.	Ref.	Ref.
Obligatory Secondary studies	−0.0742 (0.0455)	0.103	−0.0472 (0.027)	0.080
Occupational training or pre-university studies	−0.1419 (0.0542)	0.009	−0.0853 (0.0317)	0.007
University studies	−0.2043 (0.0662)	0.002	−0.1149 (0.0381)	0.003
High social class (SC)	Ref.	Ref.	Ref.	Ref.
Medium-high SC	0.2088 (0.0583)	0.000	0.1126 (0.0329)	0.001
Medium-low SC	0.3629 (0.0606)	0.000	0.2043 (0.0345)	0.000
Low social class	0.4335 (0.0688)	0.000	0.2497 (0.0396)	0.000
Intercept	−1.728 (0.1005)	0.000	−1.0266 (0.0567)	0.000
Pseudo R^2^	0.0815		0.0818	
N	22,450		22,450	

As for socioeconomic factors, regarding education level, the results show that in comparison with the reference category (primary studies or less), individuals with secondary studies have a significantly lower probability of visiting a public GP only for the probit model (*p* < 0.10). However, individuals with occupational training and individuals with university studies have a significantly lower probability of visiting a public GP for both models (*p* < 0.01). A comparison of the predicted probabilities fixing values for the relevant categories of the socioeconomic variables is presented in Table 3. Predicted probability for individuals with primary studies or less is 25.9% for binomial logit and 26.0% for binomial probit. Predicted probability for occupational training is 23.5% (for both binomial logit and probit) and predicted probabilities for those with university studies are 22.5% for binomial logit and 22.7% for binomial probit. Coefficients and predicted margins show, therefore, a clear gradient as education level decreases.

**Table 3:** Predictive margins of visiting the public general practitioner by socioeconomic variables

	Binomial Logit	Binomial Probit	Multinomial Logit	Multinomial Probit
Inactive	0.2557 (ref)	0.2558 (ref)	0.2562 (ref)	0.2562 (ref)
Employed	0.2302***	0.2307***	0.2302***	0.2305***
Unemployed	0.2512	0.251	0.2522	0.2515
Primary studies or less	0.2592 (ref)	0.2596 (ref)	0.2579 (ref)	0.2589 (ref)
Obligatory Secondary studies	0.2463	0.2457*	0.2466	0.2461
Occupational training or pre-university studies	0.2349***	0.2348***	0.2347	0.2349
University studies	0.2248***	0.2265***	0.2248	0.2271
High social class	0.2027 (ref)	0.2044 (ref)	0.2053 (ref)	0.2069 (ref)
Medium-high social class	0.2357***	0.2351***	0.2358***	0.2353**
Medium-low social class	0.2621***	0.2619***	0.2631***	0.2631***
Low social class	0.2748***	0.2758***	0.2759***	0.2768***

*** Significant coefficient at 1%, ** Significant coefficient at 5%, * Significant coefficient at 10%

Regarding social class, as compared with high social class, the lower the social class, the higher the probability of visiting a public GP (*p* < 0.01 for all coefficients in both binomial models), showing a clear gradient. While individuals in the highest social class have a predicted probability to visit a public GP of 20.3% (for binomial logit) and 20.4% (for binomial probit), individuals belonging to the low social class have 27.5 and 27.6%, respectively (Table 3). Finally, being employed (as compared with being inactive) is also negatively related to the probability of visiting a public GP both for the binomial logit and probit models (*p* < 0.01). Regarding demographic variables, older adults (aged more than 64) and females are more likely to visit a public GP.

With respect to the comprehensive approach, estimates for the multinomial logit and probit models of the public GP equation are presented in Table 4 (results for the equations of the rest of health care services can be seen in Table 6 of the Appendix). As with the binomial case, sign and statistical significance of coefficients for both models are similar. Regarding health care need, individuals with worse self-reported health states are more likely to visit a public GP with a gradient as health state worsens. Also, those with a chronic disease have a higher probability of visiting a public GP.

**Table 4:** Estimates of multinomial logit and probit models

Public GP equation^a^	Multinomial logit		Multinomial probit	
	Coefficient (se)	*p*-value	Coefficient (se)	*p*-value
Female	0.1642 (0.0343)	0.000	0.1396 (0.0271)	0.000
Age 15–34	Ref.	Ref.	Ref.	Ref.
Age 35–44	−0.066 (0.0659)	0.317	−0.0469 (0.0501)	0.349
Age 45–54	− 0.0554 (0.0649)	0.393	−0.041 (0.0497)	0.410
Age 55–64	0.0934 (0.0637)	0.143	0.0737 (0.0495)	0.137
Age more than 64	0.2698 (0.0681)	0.000	0.2199 (0.0535)	0.000
Health very good	Ref.	Ref.	Ref.	Ref.
Health good	0.3412 (0.0578)	0.000	0.2677 (0.0429)	0.000
Health fair	1.0196 (0.0646)	0.000	0.8427 (0.0494)	0.000
Health bad	1.5223 (0.082)	0.000	1.2554 (0.0638)	0.000
Health very bad	1.6227 (0.1228)	0.000	1.3361 (0.0966)	0.000
Chronics	0.7419 (0.0489)	0.000	0.5772 (0.0367)	0.000
Inactive	Ref.	Ref.	Ref.	Ref.
Employed	−0.2053 (0.0534)	0.000	−0.1642 (0.0419)	0.000
Unemployed	−0.0651 (0.0675)	0.335	−0.0579 (0.0534)	0.278
Primary studies or less	Ref.	Ref.	Ref.	Ref.
Obligatory Secondary studies	−0.0032 (0.0474)	0.946	−0.0026 (0.0382)	0.946
Occupational training or pre-university studies	−0.0502 (0.0563)	0.372	−0.0334 (0.0446)	0.453
University studies	−0.0406 (0.0685)	0.553	−0.0053 (0.0532)	0.920
High social class (SC)	Ref.	Ref.	Ref.	Ref.
Medium-high SC	0.1636 (0.0599)	0.006	0.1053 (0.0456)	0.021
Medium-low SC	0.2884 (0.0623)	0.000	0.2012 (0.0479)	0.000
Low social class	0.3396 (0.0709)	0.000	0.2446 (0.0553)	0.000
Intercept	−2.3676 (0.0991)	0.000	−1.8932 (0.0754)	0.000
Pseudo R^2^	0.0895			
N	22,450		22,450	

^a^ Not visiting any health services as reference. This table only shows the coefficients corresponding to the public general practitioner (GP) equation. Private GP, public and private specialist equations are shown in an Appendix

Regarding socioeconomic factors, social class is also negatively related to the probability of visiting a public GP. Results for the multinomial models (Table 3) indicate that individuals belonging to the high social class have a 20.5% predicted probability to visit a public GP for the multinomial logit (20.7% for the multinomial probit) whilst individuals belonging to the low social class have on average a 27.6% probability for the multinomial logit (27.7% for the multinomial probit). This difference of seven percentage points -that results to be statistically significant for both models- is quite similar to that obtained through the binomial models. However, as can be seen in Table 4, education level no longer influences the probability of visiting a public GP for both multinomial logit and probit models. In addition, the negative effect of being employed on the probability of visiting a GP also remains in this comprehensive approach.

To test the null hypothesis of independence of irrelevant alternatives (IIA), we conducted the Small–Hsiao tests [16]. None of the five tests undertaken by omitting each alternative was significant (*P* > 0.1), indicating no evidence of violation of the independence of irrelevant alternatives assumption. So, both the multinomial logit (that assumes IIA) and the multinomial probit model (that does not require the fulfilment of the IIA assumption) are valid to undertake this comprehensive approach.

One alternative way to proceed would have been to drop the 12.1% of individuals who reported having visited other doctors different from a public GP in their last visit and follow a compartmentalised approach with the remaining individuals of the sample. Estimates for the probit model with sample selection are shown in Table 5. The correlation coefficient (*rho*) is statistically different from zero (*p* < 0.01), indicating the presence of selection bias. In addition, sign, magnitude and t-ratios of coefficients of the probit model with selection are quite different to those of the simple probit estimation. Thus, we can reject the null hypothesis that there is no selection bias if we drop those observations that instead of using public GP services, have used specialist services or private GP services.

**Table 5:** Estimates of Probit model with sample selection

Public GP equation	Probit equation		Selection equation	
	Coefficient (se)	*p*-value	Coefficient (se)	*p*-value
Female	0.1081 (0.0186)	0.000	−0.0767 (0.0227)	0.001
Age 15–34	Ref.	Ref.	Ref.	Ref.
Age 35–44	−0.0215 (0.0339)	0.525	−0.023 (0.0409)	0.574
Age 45–54	−0.0112 (0.0337)	0.739	−0.0342 (0.0408)	0.401
Age 55–64	0.0488 (0.0337)	0.148	−0.0073 (0.0413)	0.861
Age more than 64	0.1073 (0.0368)	0.004	0.0914 (0.0451)	0.043
Health very good	Ref.	Ref.	Ref.	Ref.
Health good	0.2163 (0.0287)	0.000	−0.2048 (0.0365)	0.000
Health fair	0.6757 (0.0335)	0.000	−0.5386 (0.0418)	0.000
Health bad	1.0275 (0.0441)	0.000	−0.791 (0.0514)	0.000
Health very bad	1.0957 (0.0676)	0.000	−0.8163 (0.0737)	0.000
Chronics	0.43 (0.0252)	0.000	−0.2672 (0.0309)	0.000
Inactive	Ref.	Ref.	Ref.	Ref.
Employed	−0.135 (0.0286)	0.000	0.1467 (0.0345)	0.000
Unemployed	−0.0699 (0.0366)	0.056	0.0883 (0.045)	0.050
Primary studies or less	Ref.	Ref.	Ref.	Ref.
Obligatory Secondary studies	0.046 (0.0264)	0.082	−0.1637 (0.0337)	0.000
Occupational training or pre-university studies	0.0486 (0.0311)	0.117	−0.2133 (0.0383)	0.000
University studies	0.1371 (0.0385)	0.000	−0.3884 (0.0431)	0.000
High social class (SC)	Ref.	Ref.	Ref.	Ref.
Medium-high SC	0.0084 (0.0321)	0.794	0.115 (0.0344)	0.001
Medium-low SC	0.0343 (0.0352)	0.329	0.2036 (0.0379)	0.000
Low social class	0.05 (0.0406)	0.217	0.2604 (0.0462)	0.000
Double coverage			−0.4081 (0.0302)	0.000
Intercept	−1.1554 (0.0564)	0.000	1.7604 (0.0627)	0.000
rho	−0.8660 (0.0537)	0.000		
N	19,727		22,450	

## Discussion

Horizontal equity in access to public general practitioner (GP) services by socioeconomic group has been addressed econometrically in the related literature by testing the statement “equal probability of using public GP services for equal health care needs, regardless of socioeconomic status” using healthcare surveys that have information on utilisation, health status and other socioeconomic and demographic characteristics of individuals. In this research, we have claimed that there are two different approaches that could lead to different conclusions.

One is the compartmentalised approach that has been the common way to address this issue in the literature. Under this alternative, if the respondent reports that she visited a public GP, then it counts as 1, otherwise it counts as zero. Our results to this approach -applying binomial logit and probit models to data for the Spanish National Health Survey 2016–17-, indicate that the lower the education level and the lower the social class, the higher the probability of visiting a public GP, thus showing a pro-lower socioeconomic group horizontal inequity in access, in line with a great part of the related literature [1–7]. Other related studies show no evidence of horizontal inequity by socioeconomic status or sometimes inequity favouring the most advantaged [8–10]. The latter studies consider overall (public and private) GP services. The extent to which this difference can be partly explained by the difference in the definition of the dependent variable (public versus overall GP services), by the consideration of different set of socioeconomic covariates, by other methodological issues, by the differences in the organization and provision of health care in the countries analysed (Spain, Belgium, Norway, Sweden or the UK) or simply by a different empirical evidence, is not known and would involve further research. With this caveat in mind, what is common to all these studies is that they share the compartmentalised approach.

As it has been highlighted, it may be the case that the respondent’s last visit was not a public GP but was say a specialist doctor or a private GP. Binomial econometric models would wrongly consider such visits as zeros (i.e. non-utilisation), thus potentially obtaining biased conclusions regarding horizontal equity in access. In order to test for this potential bias, we undertook a comprehensive approach through multinomial logit and probit models to account for utilisation different from the public GP in the last visit. On the one hand, conclusions regarding social class remain very similar to those of the binomial logit and probit models: the lower the social class, the higher the probability of visiting a public GP, also in line with some of the revised previous studies [1, 2, 4]. In addition, the lower propensity to visit a public GP of those employed (with respect to those inactive individuals) found in the binomial approach, remains under the multinomial approach (a relatively higher opportunity cost of time may help to explain this negative effect). However, interestingly, we find that the education effect found in the compartmentalised approach, by which individuals with lower education level have a higher propensity to visit a public GP (a finding which would be consistent with one of the predictions of the Grossman model, in which individuals with higher education level are more efficient at producing health, and therefore they need to use health care services less [17]), disappears under the comprehensive approach. The lack of evidence to reject the independence of irrelevant alternative hypothesis indicates that both multinomial logit and probit models are appropriate. The fact that both models give similar results reinforces the strength of our conclusions. This evidence contradicts previous findings regarding the effect of education on access to public GP services [1–7] but is in line with results obtained in other studies [8–10]. One possible explanation is that individuals with higher education levels (as compared with those with primary studies or less) instead of using public GP services, are using relatively more public specialist services (and/or other private specialist care or GP services) either as substitute or complementary, thus, not losing (if not increasing) access to the public health care system. For example, Negrín et al. show evidence that suggests that the relatively higher propensity to combine public and private specialist visits of those with higher education levels, is related to a complementary use of private health care services in order to reduce waiting times in the public sector specialist care [18]. And our results regarding the multinomial logit and probit models seem to reinforce such hypothesis, according to the positive and significant coefficients of the education categories for the corresponding three equations (see Table 6 in the Appendix).

Another possible approach would have been to drop those individuals who reported to be in another part of the health system and then undertake a binomial logit/probit model to estimate the probability of visiting a public GP. However, if as a consequence of dropping that part of the sample, a problem of selection bias arises, then this approach is not reliable. This is what actually happened with our database.

To conclude, although we have undertaken our analysis with the same survey under the same health care system (so any bias encountered can be attributed to the differences in approaches) we must be cautious when generalising our results to other contexts. However, we strongly suggest that it is appropriate to undertake a comparison between both approaches. If the binomial/compartmentalised approach -which, as compared with the multinomial approach, is more straightforward to interpret and allows for other outputs like the construction of inequity indices- gives results that are not different from those of the multinomial/comprehensive approach, then this comparison exercise adds robustness to their results. On the other hand, when a significantly different result is obtained, a comprehensive approach seems to be more appropriate. If that is the case, the conclusion reached by the binomial approach about horizontal inequity would be incorrect due to the fact that individuals using other health care services are wrongly considered as non-users and this situation should not be regarded as inequitable.

Regarding the paper’s limitations, four points must be made. First, it should be noted that our main database (Spanish National Health Survey) does not have enough information to analyse horizontal equity taking into account the intensity of use (i.e. number of GP visits), as we only have the relevant information for the very last visit. A comprehensive approach that considers the number of visits/contacts would be much more demanding information-wise and probably very difficult to undertake. Second, conclusions on equity in access to public GP visits must be taken with caution as the health survey used only has information on doctors’ visits in the past four weeks. A longer reference period might change the conclusions, although it must also be said that we would then face a greater risk of recall bias. Third, health care need is not the same for different health services (general practitioner vs. specialist). However, the available data do not allow for a distinction in the health needs of different services. In any case, we consider that self-assessed health and self-reported chronic diseases are good predictors of demand for public GP visits (whilst for specialist visit, particularly those of the public health care system, a good indicator of health care need would be the specialist appointment itself, insofar as it must have been indicated previously by a doctor (GP or specialist). Finally, the comprehensive approach assumes that the five options are available for all the sample. Civil servants in Spain have the right to choose annually between the public provider and a private insurance provider. Thus, those civil servants who have chosen a private health care provider should not have access to public services. However, they actually do have access to public services, possibly because of the little control over this fraudulent double coverage (Sanchez-Bayle and Beiras [19] and Rodríguez and Stoyanova [20]). In addition, it must be noted that this group represents the 3.05% of the whole sample.

## Conclusion

The conclusion -supported by a considerable part of the compartmentalised approach literature- that access to public GP services favours the lower socioeconomic groups is debatable, at least regarding the effect of education level. The multinomial analysis suggests that the social class effect remains, but the education level effect that resulted from the binomial analysis disappears when the utilisation of other health care services (apart from public GP health care services) is considered within the analysis. An analysis of horizontal equity in access to a particular health care service undertaken following a compartmentalised approach should be compared with a comprehensive approach in order to test that there is no bias as a consequence of considering as zeros the utilisation of other types of health care.

## Data Availability

The data base is available in open in the following website: http://www.mscbs.gob.es/estadEstudios/estadisticas/encuestaNacional/encuesta2017.htm

## Appendix

**Table 6:** Estimates of the multinomial logit and probit (rest of equations)

Private GP equation	Multinomial logit		Multinomial probit	
	Coefficient (se)	*p*-value	Coefficient (se)	*p*-value
Female	0.0761 (0.1136)	0.503	0.0649 (0.058)	0.263
Age 15–34	Ref.	Ref.	Ref.	Ref.
Age 35–44	0.2581 (0.2129)	0.226	0.1109 (0.1052)	0.292
Age 45–54	0.3061 (0.2144)	0.153	0.1249 (0.1063)	0.240
Age 55–64	0.2628 (0.2205)	0.233	0.1213 (0.1093)	0.267
Age more than 64	0.6236 (0.2326)	0.007	0.3058 (0.1169)	0.009
Health very good	Ref.	Ref.	Ref.	Ref.
Health good	0.2652 (0.167)	0.112	0.1918 (0.0853)	0.025
Health fair	0.7346 (0.2007)	0.000	0.5703 (0.1027)	0.000
Health bad	1.3745 (0.264)	0.000	1.0034 (0.1351)	0.000
Health very bad	0.3553 (0.6107)	0.561	0.5842 (0.2751)	0.034
Chronic	0.4233 (0.1458)	0.004	0.3269 (0.0746)	0.000
Inactive	Ref.	Ref.	Ref.	Ref.
Employed	−0.2397 (0.1814)	0.186	−0.1546 (0.0914)	0.091
Unemployed	−0.308 (0.2628)	0.241	−0.1659 (0.128)	0.195
Primary studies or less	Ref.	Ref.	Ref.	Ref.
Obligatory Secondary studies	0.6511 (0.2171)	0.003	0.3318 (0.1006)	0.001
Occupational training or pre-university studies	1.2709 (0.2126)	0.000	0.6176 (0.1022)	0.000
University studies	1.6011 (0.2228)	0.000	0.8323 (0.1078)	0.000
High social class (SC)	Ref.	Ref.	Ref.	Ref.
Medium-high SC	−0.3199 (0.1419)	0.024	−0.176 (0.0751)	0.019
Medium-low SC	−1.2271 (0.203)	0.000	−0.5916 (0.0957)	0.000
Low social class	−1.309 (0.2946)	0.000	−0.6203 (0.1323)	0.000
Intercept	−5.1067 (0.327)	0.000	−3.2551 (0.1568)	0.000
Public Specialist equation	Multinomial logit		Multinomial probit	
	Mean (se)	p-value	Mean (se)	p-value
Female	0.2037 (0.0537)	0.000	0.1492 (0.035)	0.000
Age 15–34	Ref.	Ref.	Ref.	Ref.
Age 35–44	−0.0379 (0.1016)	0.709	−0.0167 (0.0645)	0.795
Age 45–54	0.0066 (0.0985)	0.946	0.0079 (0.0635)	0.901
Age 55–64	0.0169 (0.0979)	0.863	0.0283 (0.0636)	0.657
Age more than 64	−0.2071 (0.1066)	0.052	−0.096 (0.0692)	0.166
Health very good	Ref.	Ref.	Ref.	Ref.
Health good	0.544 (0.104)	0.000	0.3522 (0.0604)	0.000
Health fair	1.4998 (0.112)	0.000	1.0405 (0.0672)	0.000
Health bad	2.3166 (0.128)	0.000	1.6332 (0.0812)	0.000
Health very bad	2.4384 (0.1699)	0.000	1.7281 (0.115)	0.000
Chronic	0.742 (0.0815)	0.000	0.529 (0.0493)	0.000
Inactive	Ref.	Ref.	Ref.	Ref.
Employed	−0.28 (0.0796)	0.000	−0.1959 (0.0527)	0.000
Unemployed	−0.1337 (0.1005)	0.183	−0.0931 (0.0672)	0.166
Primary studies or less	Ref.	Ref.	Ref.	Ref.
Obligatory Secondary studies	0.2421 (0.0753)	0.001	0.169 (0.0497)	0.001
Occupational training or pre-university studies	0.224 (0.0883)	0.011	0.1619 (0.0579)	0.005
University studies	0.3999 (0.1038)	0.000	0.2912 (0.0674)	0.000
High social class (SC)	Ref.	Ref.	Ref.	Ref.
Medium-high SC	0.072 (0.0888)	0.418	0.0362 (0.0569)	0.525
Medium-low SC	0.0989 (0.0936)	0.291	0.0527 (0.0602)	0.382
Low social class	−0.011 (0.1098)	0.920	−0.0169 (0.0711)	0.812
Intercept	−3.7594 (0.1603)	0.000	−2.6967 (0.0993)	0.000
Private Specialist equation	Multinomial logit		Multinomial probit	
	Coefficient (se)	p-value	Coefficient (se)	p-value
Female	0.3034 (0.0831)	0.000	0.1877 (0.047)	0.000
Age 15–34	Ref.	Ref.	Ref.	Ref.
Age 35–44	0.1178 (0.1391)	0.397	0.0485 (0.0798)	0.544
Age 45–54	0.0272 (0.1426)	0.849	0.013 (0.0812)	0.873
Age 55–64	−0.1075 (0.1495)	0.472	−0.0495 (0.0843)	0.557
Age more than 64	−0.0627 (0.1624)	0.700	−0.0252 (0.0918)	0.783
Health very good	Ref.	Ref.	Ref.	Ref.
Health good	0.3097 (0.1233)	0.012	0.2139 (0.0695)	0.002
Health fair	1.1203 (0.1434)	0.000	0.7801 (0.0816)	0.000
Health bad	1.4544 (0.2017)	0.000	1.0387 (0.1125)	0.000
Health very bad	1.7588 (0.2802)	0.000	1.2381 (0.1623)	0.000
Chronic	0.507 (0.1059)	0.000	0.3954 (0.0602)	0.000
Inactive	Ref.	Ref.	Ref.	Ref.
Employed	−0.2145 (0.1251)	0.086	−0.1409 (0.0708)	0.047
Unemployed	−0.4884 (0.1866)	0.009	−0.268 (0.1007)	0.008
Primary studies or less	Ref.	Ref.	Ref.	Ref.
Obligatory Secondary studies	0.701 (0.1548)	0.000	0.3559 (0.0789)	0.000
Occupational training or pre-university studies	0.9879 (0.1598)	0.000	0.5083 (0.0835)	0.000
University studies	1.5129 (0.1642)	0.000	0.8333 (0.0881)	0.000
High social class (SC)	Ref.	Ref.	Ref.	Ref.
Medium-high SC	−0.5003 (0.1044)	0.000	−0.2938 (0.062)	0.000
Medium-low SC	−1.1813 (0.1385)	0.000	−0.6321 (0.0749)	0.000
Low social class	−1.3547 (0.2003)	0.000	−0.715 (0.1021)	0.000
Intercept	−4.2553 (0.231)	0.000	−2.8639 (0.1248)	0.000
